# Purification of a Novel Anti-VEGFR2 Single Chain Antibody Fragment and Evaluation of Binding Affinity by Surface Plasmon Resonance

**DOI:** 10.15171/apb.2019.008

**Published:** 2019-02-21

**Authors:** Shirafkan Kordi, Mohammad Rahmati-Yamchi, Mehdi Asghari Vostakolaei, Abolfazl Barzegari, Jalal Abdolalizadeh

**Affiliations:** ^1^Immunology Research Center, Tabriz University of Medical Sciences, Tabriz, Iran.; ^2^Department of Medical Biotechnology, Faculty of Advanced Medical Sciences, Tabriz University of Medical Sciences, Tabriz, Iran.; ^3^Biotechnology Research Center, Tabriz University of Medical Sciences, Tabriz, Iran.; ^4^Student Research Committee, Tabriz University of Medical Sciences, Tabriz, Iran.; ^5^Research Centre for Pharmaceutical Nanotechnology, Tabriz University of Medical Sciences, Tabriz, Iran.; ^6^Drug Applied Research Center, Tabriz University of Medical Sciences, Tabriz, Iran.; ^7^Paramedical Faculty, Tabriz University of Medical Sciences, Tabriz, Iran.

**Keywords:** Human VEGFR2, Monoclonal Antibody, Phage Display, Purification, ScFv, Surface Plasmon Resonance

## Abstract

***Purpose:*** The single-chain variable fragment (scFv) domain of antibodies is now considered as one of the therapeutic tools that can be produced by phage display technology (PDT). Antibody purification is one of the most important steps in antibodies production. The aim of study was purification and characterization of anti-VEGFR2 scFv antibody fragments.

***Methods:*** After the coating of vascular endothelial growth factor receptor 2 (VEGFR2) peptide in ELISA microplates, the phage display library of Tomlinson was used for antibody isolation. The targeted scFv was purified by chromatography using a zeolite-based column. The purity and functional assessment of purified scFv were evaluated by sodium dodecyl sulfate polyacrylamide gel electrophoresis (SDS-PAGE) and western blotting techniques, respectively. Affinity binding was evaluated by surface plasmon resonance (SPR).

***Results:*** The desired scFv was selected after four stages of biopanning. SDS-PAGE analysis showed a 28 kDa scFv with high purity (>90%). The western bloting analysis confirmed the binding of produced scFv antibody to the desired peptide. The affinity binding of scFv antibody analyzed by SPR was about 60 μM.

***Conclusion:*** In this study, the novel scFv antibody against VEGFR2 peptide was purified by chromatography column containing zeolite. Based on our findings the produced antibody may be applied for diagnosis or targeting of VEGFR2 in antibody-based therapy strategies.

## Introduction

Despite the variety of cancer treatments, antibodies are now effective in the treatment and prevention of cancer progression.^1^ They can invade cancer cells by targeting a specific tumor antigen with minimal damage to normal cells. The first anticancer monoclonal antibodies (mABs) were murine antibodies produced by the hybridoma technique.^2^ Although these antibodies had a high affinity to the target antigen, they were associated with a host immune response. The chimeric antibodies were introduced to reduce the host immune response that constitutes the murine variable region and the human constant region, whose immunogenicity is less than the murine type. Human antibodies were made to minimize immunogenicity, of which only the complementarity-determining regions (CDRs) were of the mouse type.^3^ At present, a fully human single-chain variable fragment (scFv) antibody can be made using phage display without the problem of immunogenicity.

The vascular endothelial growth factor (VEGF) family and its most important receptor, VEGFR2, are involved in angiogenesis.^4^ Solid tumors require angiogenesis for growth and metastasis^5^ and VEGFR2 is one of the important antigens that can be targeted with high-affinity antibodies.^6^

Antibody purification involves steps employed in the separation of the target protein from the protein mixture. Recent advances in the design of chromatography columns for the purification of biomolecules have made it possible to carry out more precise experiments and result to a general pattern for industrialization of the purification process.^7,8^ Affinity chromatography is one of the most commonly used methods for the first part of the purification of antibody fragments.^9-11^

Nowadays, the application of surface plasmon resonance (SPR) biosensors describes significant information and the main details about biomolecular interactions.^12,13^ The SPR phenomenon is widely used as a technology for studying the interactions between biomolecules like antigen-antibody, drug-albumin, nucleotides, cell- drug and the detection of cell surface markers.^14-17^ The use of an SPR based method for screening the kinetics and affinity parameters in small specific antigen-antibody interactions is more useful than immune-enzymatic techniques like ELISA due to the analysis of biomolecular interactions in real-time and label free form.^18,19^

The present study aimed to purify anti-VEGFR2 scFv antibody followed by characterization through SPR.

## Materials and Methods

### Selection of scFv by biopanning

The scFv antibody library, Tomlinson I and J, were purchased from MRC Laboratory (Cambridge, UK). For the selection of specific phage antibody clones, VEGFR-2 synthetic peptide firstly immobilized onto wells. Then, the coated wells were blocked with blocking buffer (5% skim milk in PBS). An aliquot (500 µL) of the phage antibody library was added into each wells and incubated at room temperature (RT) for 2 hours. Unbound phages were eluted by trypsin (Sigma Chemical, St. Louis, MO, USA) and added to 1.7 mL of *Escherichia coli* TG1 in log phase (Stratagene. La Jolla, CA, USA). The eluted phages were amplified in fresh TG1 cells for the next round of panning. Overall, 4 biopanning rounds were carried out in a stepwise reduced concentration of VEGFR-2 synthetic peptide. The amount of coated peptide was reduced serially from 100 μg/well to 0.1 μg/well from the first to fourth steps.^20,21^

### Purification of scFv using the chromatography column containing zeolite

After selection of scFv by biopanning with phage display technology (PDT) and its expression in host, the purification of scFv antibodies was performed using zeolite (ZSM-5-Ni^+2^) using the BOECO Rotator Multi Bio RS-24.^22^

For the synthesis of zeolite, 50 mM sodium hydroxide and 6 mM sodium aluminate were dissolved in ion-free water and then mixed with sufficient amount of sodium silicate solution (25%) with vigorous stirring for 12 hours. The mixture was placed at 180°C for 32 hours. After filtration process, it was washed with distilled water and dried at 90°C. The stabilization of Ni + 2 ion on zeolite was carried out using ion exchange method of the liquid phase by a saturation solution of Ni (NO3)_2_ 6H2O in RT for 72 hours.

At first, 20 mg of zeolite was equilibrated for 20 minutes as a constant phase with 50 mM Na_2_HPO_4_, 0.5M of NaCl, pH 8 buffer. The centrifugation was done for 5 minutes at 5000 g and the supernatant was discarded. Next, 1ml of protein mixture of periplasmic and cytoplasmic extraction was dialyzed and added to zeolites. After centrifugation, the supernatant was removed. The washing process was carried out using washing buffer (50 mM of Na_2_HPO_4_, 0.5M of NaCl, and 20 mM of imidazole, pH 8) for 20 minutes. The elution process was performed by elution buffer (50 mM of Na2HPO4, 0.5 M of NaCl, 500 mM imidazole pH 8).

### SDS-PAGE and western blotting Analysis

To evaluate the size and purification of scFv fragments, the products were analyzed by sodium dodecyl sulfate polyacrylamide gel electrophoresis (SDS-PAGE) under reduced condition. The bands were observed after staining with Coomassie Blue.

The western blotting analysis was designed to functional assessment of produced scFv antibody fragments. After SDS-PAGE, the electrophoresis profile was blotted onto polyvinylidene fluoride (PVDF) membrane (Millipore, Billerica, MA, USA) using a semidry transfer device (Amersham Biosciences, Freiburg, Germany). After blocking with 5% skim milk, the membrane was incubated with mouse anti-Myc (Santa Cruz Biotechnology, Santa Cruz, CA, USA) and HRP-conjugated anti-mouse IgG (Santa Cruz Biotechnology, Santa Cruz, CA, USA) antibodies. Finally the enhanced chemiluminescence substrate (Amersham Biosciences, Freiburg, Germany) was used to observe the bands.

### SPR measurements

Measurements were done using a multi-parametric SPR (MP-SPR Navi 210A, BioNavis Ltd, Finland) for kinetic and antigen-antibody binding affinity. This study was probed in fixed angle mode after initial scan of gold surface mode with a flow rate of 25 µl min^−1^ at fixed temperature of 25°C. The wavelength of laser source for exciting the surface plasmonson at the dielectric gold interface was 670 nm. SPR-Navi 210A gold chips were prepared from BK7- glass slide near 250 mm^2^ surface area that sputtered 50 nm gold layers on it.

### Modification of gold chip with carboxylic groups

First, each gold chip was put in boiling solution of ammonia (NH_4_OH, 30% w/v) and hydrogen peroxide (H_2_O_2_, 30% w/v) in Milli-Q-water for about 15 minutes at 90°C. Then, the gold chips were washed with distilled water and ethanol solution and finally the stream of nitrogen was passed on it. In order to formation of carboxyl functional group on gold surface, a pure gold chip was placed in the solution containing 5 mM 11-mercaptoundecanoic acid (MUA) and distilled water in ratio of 7 to 3 at room temperature. After 24 hours, gold chips were modified with MUA molecules and washed several times with running buffer and ethanol and then dried using the stream of nitrogen.

### Antibody immobilization through covalent attachment

After reaching a steady baseline and straight- line after 20 minutes, the MUA modified gold surface was washed with NaCl (2 M) and NaOH (0.1 M) for 4 minutes. A usual protein immobilization method was done by using covalent interaction. To activate the carboxylic surface, EDC: NHS 1:1 (NHS 0.05 M + EDC 0.2 M) solution activated the carboxylic groups surface for 4 minutes. The activated slides were immediately used for injection of antibody solution (100 µg/mL) in PBS buffer with pH 6.5 at flow rate of 10 µL/min for 8 minutes. Then, ethanolamine-HCl (1.0 M) solution was flowed to the modified groups to inactivate the non-immobilized surface on gold chip.

### Kinetic analysis of antibody-antigen interaction

Various concentration of peptide (0-10 µM) in PBS buffer, pH 7.5, were injected with a flow rate of 25 µl/min for 2 minutes. Since antibody was immobilized at one of flow cells, one of them was applied for using as reference. For correcting bulk effects, the response of a blank reference spot was subtracted from the response of the ligand surface. TraceDrawer^TM^ for SPR Navi^TM^ was applied for calculation of affinity and kinetic of the measured interaction. Before calculation, the TraceDrawer data is extracted with SPR Navi^TM^ data viewer software.

## Results and Discussion

### Screening and selecting scFv by biopanning

**Figure 1:**
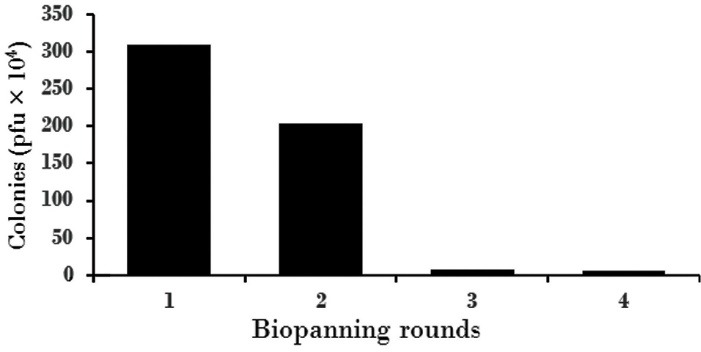
Biopanning with an antibody phage-display library against VEGFR2. The isolated anti-VEGFR2 peptide phage titer based on the plaque-forming unit, biopanning was completed in four steps by isolating the specific phages.
VEGFR2: vascular endothelial growth factor receptor 2.

At first, (I + J) human scFv Tomlinson library was proliferated and used to select anti-VEGFR2 scFv. In general, the process of isolating anti- VEGFR2 specific phages was repeated four times. The number of colonies in the TYE medium was decreased in biopanning rounds. In biopanning rounds, the amount of coated peptide was ten times lower from previous step and the number of rinses was also increased. The concentration of peptidase reached 100 μg/well in the first stage and 0.1 μg/well in the fourth stage and the frequency of rinsing was increased from 3 to 10 times in the fourth stage. In this case, the specific phages are isolated in the final stages (Figure 1).

### SDS-PAGE and western blot analysis

**Figure 2:**
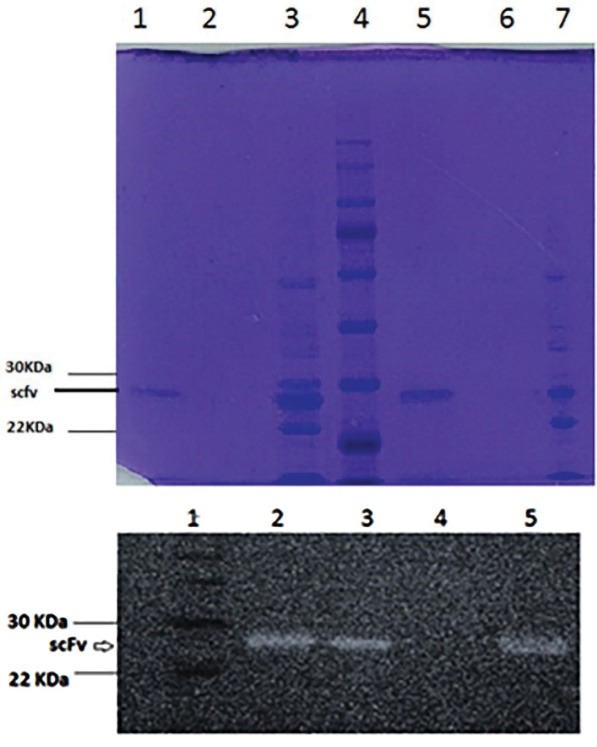
**(A)** Electrophoresis profile of scFvs against VEGFR2. The expressed scFvs were electrophoresed on 12% SDS-PAGE after purification and observed with Coomassie brilliant blue staining. Lane1: crude cytoplasmic extraction, Lane2: washing fraction (cytoplasmic extraction), Lane3: elution fraction (cytoplasmic extraction), Lane4: protein size marker, Lane5: crude periplasmic fraction, Lane6: washing fraction (periplasmic extraction) and 7- elution (periplasmic extraction). (B) In vitro binding analysis of the selected scFv against VEGFR2 using Western blot. The purified scFvs after isolation were electrophoresed on 12% SDS-PAGE, blotted on a PVDF membrane after the addition of antibodies, the protein band was finally detected using the enhanced chemiluminescence substrate. The 28 kDa band of the desired scFv is along the side of the ladder. The lanes are respectively1- ladder 2- elution from the periplasmic fraction 3- elution from the cytoplasmic fraction 4- wash of collect and 5- positive control.

The electrophoresis was done on 12% SDS-PAGE on reduced condition. The protein bands were stained with Coomassie Blue R 250. The purity of produced scFv was more than 90% with a molecular weight of 28 kDa (Figure 2A).

scFv: single-chain variable fragment, VEGFR2: vascular endothelial growth factor receptor 2, SDS-PAGE: Sodium dodecyl sulfate polyacrylamide gel electrophoresis, PVDF: polyvinylidene fluoride, kDa: Kilo Dalton.

The western blot results confirmed the binding and presence of scFv antibody fragment with a molecular weight of 28 kDa (Figure 2B).

Despite the design of various commercial resins, efforts are still under way to design new affinity chromatography resins with better performance. The zeolite resin has a porous structure with cation exchange property that can be exploited as a base for the design of affinity chromatography resin for the purification of scFv antibodies. After the biopanning and expression processes, IMAC was used for antibody purification. A comparison of the IMAC resin designed in this study with the resins used in other studies for scFv antibody purification showed the high potential of the proposed resin.^22^ The purity obtained with this method was over 90%. Sushma et al designed the IMAC resin with a copper ion stabilization on the commercial methacrylate substrate for purification of the scFv antibodies and obtained 90% purification after two-stage purification by IMAC resin and a commercial anion exchange column.^23^ In another study, the commercial column of IMAC (Pharmacia) was used to purify the scFv antibody and the purification process was fulfilled with a purification percentage of 93% and a yield of 24%.^24^ Also, Kurasawa et al used the IMAC column to purify the scFv antibody and achieved a purification percentage of 83% and a yield of 9%. The second stage of chromatography by the SEC column promoted the purification percentage up to 96%.^25^

The results of SDS-PAGE and western blotting after purification, indicate that the purification has been performed correctly and additional proteins have been removed from the purification process, and the isolated scFv has been binded to the VEGFR2 peptide.

### Immobilization of antibody through amine coupling

**Figure 3:**
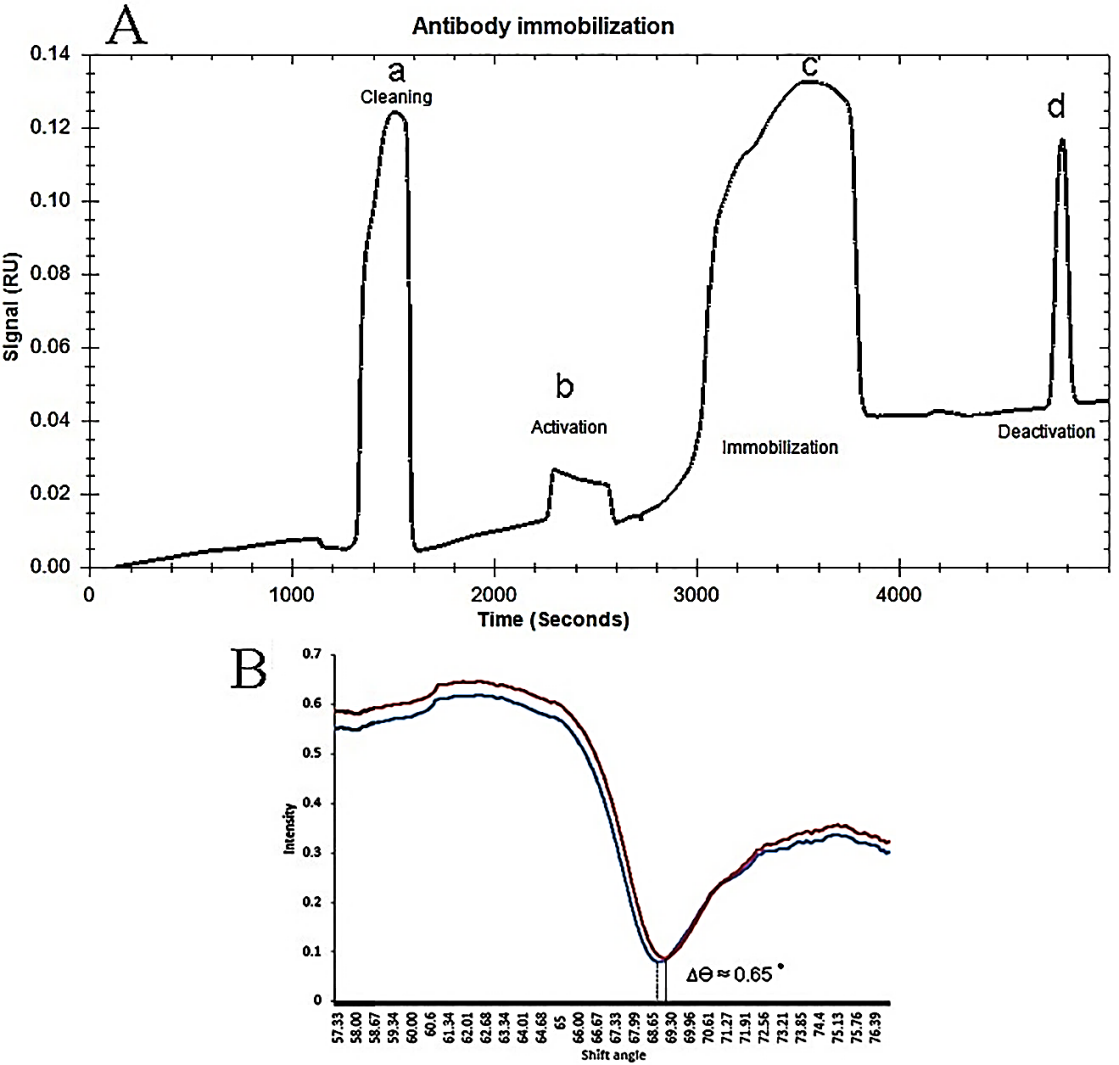
**(A)** SPR sensogram of antibody immobilization on a MUA-modified gold-sensor slide at pH 6.5 (a). Cleaning: cleaning of the MUA with NaCL. (2 M) + NaOH (0.01 M) (injection time: 4 min, flow rate: 15 µL/min (b); activation: activation of the MUA with a mixture of EDC/NHS (injection time: 4 min, flow rate: 15 (µL/min); immobilization: immobilization of 100 µg/mL antibody (injection time:8 min, flow rate: 10 µl/min); deactivation: deactivation of remaining reactive NHS esters with ethanolamine (injection time:1 min, flow rate: 15 µL/min). SPR: surface plasmon resonance. (**B)** Angle shift of surface plasmon resonance curve before (blue curve) and after (red curve) 100 µg/mL antibody immobilization on 11-mercaptoundecanoic acid functionalized gold slide surface.

In Figure 3A the sensogram of immobilization steps was shown. After carboxylic modification on gold surface by MUA, gold surface was activated via EDC/NHS and then antibody was attached onto the carboxylic groups using covalent amide binding creation as described previously. Finally, ethanolamine was used for blocking the unmodified molecules of surface. Immobilization of antibody was done in pH 6.5 of PBS buffer due to having positive charge of antibody below its isoelectric point (pI). Therefore, negative charge of carboxylic groups on MUA surface may cause to great electrostatic binding formation on the modified gold surface at pH 6.5. The level of obtained response units (RU) for successful immobilization step was near 0.03RU which the SPR curve angle shift of MUA-gold surface compared to antibody-immobilized sensor confirmed this attachment by 0.65^◦^ shifting of angle (Figure 3B). From the above results, it can be concluded that the formation of MUA SAM and antibody immobilization was completed suitably on the gold sensor surface.

### Kinetics of antibody-antigen interaction

**Figure 4:**
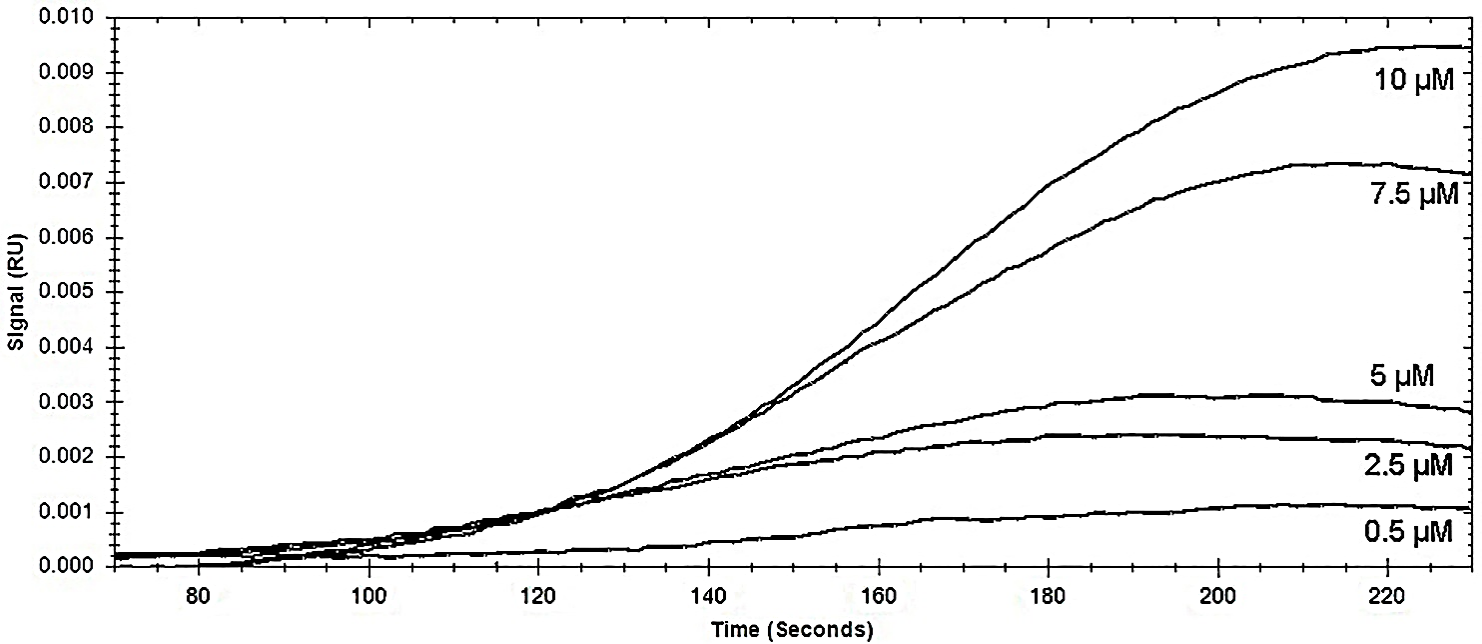
Sensogram of Ag interaction with immobilized antibody at pH 7.5. Five concentrations of Ag (0.5, 2.5, 5, 7.5 and 10 µM) have been used.

In immuno-sensors that are based on the specific recognition of Ab-Ag interactions, antibodies can be attached onto different solid surfaces by physical or chemical immobilization, covalent binding, using diverse linkages such as glutaraldehyde, carbodiimide, proteins A. Also, in calculation of kinetic constants of antigen-antibody interaction, antibody immobilization is more suitable due to screen a high number of analogs and practical and analytical points. Figure 4 shows dose-response sensograms obtained for immobilized antibody interaction with related antigen (0.5 to 10 µM), that flowed on modified surface after reference deletion. For decreasing mass transport result that lead to incorrect data, not only the antibody molecule was attached at the low amount but also the flow rate of affinity experiments set at an upper rate of 25 µL/min. The outcomes of kinetic rate constants (ka and kd) as well as equilibrium dissociation constant (KD) are summarized in Table 1 with binding model of 1:1 interaction (K_D_ = 62.8 μM). The lesser the KD the higher molecule affinity is kinetic parameters gives information how fast things happen. Association rate constant (ka) is number of Ab-Ag complex made per second and dissociation rate constant (kd) is fraction of complexes falloff per second.

**Table 1:** Kinetic measurements data of Ag-Ab interaction

**K**_a_ (1/ (M*S))	**K**_d_ **(1/S)**	**K**_D_ ** (M)**
8.7e^1^	5.4e^-3^	6.28e^-5^

K_a_: Association rate constant. K_d_: Dissociation rate constant. K_D_: Equilibrium dissociation constant.

In SPR measurements, any trivial mass and refractive index changes near the gold sensor surface, as a sensitive probe, is capable of detecting these changes by angle shifts of SPR curves in an array format of the modified gold surface.^26^ The evaluation of kinetic and affinity results suggests that scFv purification has a good quality for binding to the VEGFR2 peptide, which can even be improved and optimized by protein engineering. Our selective scFv has a moderate affinity compared to previous antibodies, but higher affinity always does not mean that it is more effective. A number of studies have shown that penetration into the tumor in scFvs with lower affinity is greater than those with high affinity.^27-29^

## Conclusion

In this study, selection of scFv antibody fragment against VEGFR2 peptide was done by biopanning using PDT. Then selected scFv was purified by modified IMAC; its purity and functional assessment were evaluated by SDS-PAGE and western blotting, respectively. Also SPR analysis was used for binding affinity of purified scFv to desired peptide.

We believe by performing other supplementary in vivo and in vitro tests, the produced scFv can be applied for diagnosis and targeting of VEGFR2 in antibody-based therapy strategies.

## Acknowledgments

These data are part of Ph. D thesis registered in Tabriz University of Medical Sciences, Iran. The authors are grateful for financial support by the Immunology Research Center of Tabriz University of Medical Sciences.

## Ethical Issues

The research project has approved by the Ethics Committee of Tabriz University of Medical Sciences with the number of 94.2-1.8.

## Conflict of Interest

The authors declare no conflict of interest.

## References

[1] Reichert JM, Rosensweig CJ, Faden LB, Dewitz MC (2005). Monoclonal antibody successes in the clinic. Nat Biotechnol.

[2] Köhler G, Milstein C (1975). Continuous cultures of fused cells secreting antibody of predefined specificity. Nature.

[3] Riechmann L, Clark M, Waldmann H, Winter G (1988). Reshaping human antibodies for therapy. Nature.

[4] Ferrara N, Gerber HP, LeCouter J (2003). The biology of vegf and its receptors. Nat Med.

[5] Takahashi S (2011). Vascular endothelial growth factor (vegf), vegf receptors and their inhibitors for antiangiogenic tumor therapy. Biol Pharm Bull.

[6] Falcon BL, Chintharlapalli S, Uhlik MT, Pytowski B (2016). Antagonist antibodies to vascular endothelial growth factor receptor 2 (vegfr-2) as anti-angiogenic agents. Pharmacol Ther.

[7] Aghebati Maleki L, Majidi J, Baradaran B, Abdolalizadeh J, Kazemi T, Aghebati Maleki A (2013). Large scale generation and characterization of anti-human cd34 monoclonal antibody in ascetic fluid of balb/c mice. Adv Pharm Bull.

[8] Sineh Sepehr K, Baradaran B, Majidi J, Abdolalizadeh J, Aghebati L, Zare Shahneh F (2013). Mass-production and characterization of anti-cd20 monoclonal antibody in peritoneum of balb/c mice. Adv Pharm Bull.

[9] Abdolalizadeh J, Nouri M, Zolbanin JM, Barzegari A, Baradaran B, Barar J (2013). Targeting cytokines: Production and characterization of anti-tnf-α scfvs by phage display technology. Curr Pharm Des.

[10] Raoufinia R, Mota A, Nozari S, Aghebati Maleki L, Balkani S, Abdolalizadeh J (2016). A methodological approach for purification and characterization of human serum albumin. J Immunoassay Immunochem.

[11] Balkani S, Shamekhi S, Raoufinia R, Parvan R, Abdolalizadeh J (2016). Purification and characterization of bovine serum albumin using chromatographic method. Adv Pharm Bull.

[12] Scarano S, Mascini M, Turner AP, Minunni M (2010). Surface plasmon resonance imaging for affinity-based biosensors. Biosens Bioelectron.

[13] Fathi F, Rahbarghazi R, Rashidi MR (2018). Label-free biosensors in the field of stem cell biology. Biosensors and Bioelectronics.

[14] Zhang Q, Zou XN, Chu LQ (2018). Surface plasmon resonance studies of the hybridization behavior of DNA-modified gold nanoparticles with surface-attached DNA probes. Plasmonics.

[15] Fathi F, Rezabakhsh A, Rahbarghazi R, Rashidi MR (2017). Early-stage detection of ve-cadherin during endothelial differentiation of human mesenchymal stem cells using spr biosensor. Biosens Bioelectron.

[16] Pope ME, Soste MV, Eyford BA, Anderson NL, Pearson TW (2009). Anti-peptide antibody screening: Selection of high affinity monoclonal reagents by a refined surface plasmon resonance technique. J Immunol Methods.

[17] Fathi F, Mohammadzadeh Aghdash H, Sohrabi Y, Dehghan P, Ezzati Nazhad Dolatabadi J (2018). Kinetic and thermodynamic studies of bovine serum albumin interaction with ascorbyl palmitate and ascorbyl stearate food additives using surface plasmon resonance. Food Chem.

[18] Khajeh S, Tohidkia MR, Aghanejad A, Mehdipour T, Fathi F, Omidi Y (2018). Phage display selection of fully human antibody fragments to inhibit growth-promoting effects of glycine-extended gastrin 17 on human colorectal cancer cells. Artif Cells Nanomed Biotechnol.

[19] Sharifi M, Ezzati Nazhad Dolatabadi J, Fathi F, Zakariazadeh M, Barzegar A, Rashidi M (2017). Surface plasmon resonance and molecular docking studies of bovine serum albumin interaction with neomycin: Kinetic and thermodynamic analysis. BioImpacts.

[20] Abdolalizadeh J, Nouri M, Zolbanin JM, Baradaran B, Barzegari A, Omidi Y (2012). Downstream characterization of anti-tnf-α single chain variable fragment antibodies. Hum Antibodies.

[21] Aghebati-Maleki L, Younesi V, Baradaran B, Abdolalizadeh J, Motallebnezhad M, Nickho H (2017). Antiproliferative and apoptotic effects of novel anti-ror1 single-chain antibodies in hematological malignancies. SLAS Discov.

[22] Mesgari Shadi A, Sarrafzadeh MH, Divband B, Barar J, Omidi Y (2018). Batch adsorption/desorption for purification of scfv antibodies using nanozeolite microspheres. Microporous Mesoporous Mater.

[23] Sushma K, Vijayalakshmi MA, Krishnan V, Satheeshkumar PK (2011). Cloning, expression, purification and characterization of a single chain variable fragment specific to tumor necrosis factor alpha in escherichia coli. J Biotechnol.

[24] Freyre FM, Vázquez JE, Ayala M, Canaán Haden L, Bell H, Rodríguez I (2000). Very high expression of an anti-carcinoembryonic antigen single chain fv antibody fragment in the yeast pichia pastoris. J Biotechnol.

[25] Kurasawa JH, Shestopal SA, Jha NK, Ovanesov MV, Lee TK, Sarafanov AG (2013). Insect cell-based expression and characterization of a single-chain variable antibody fragment directed against blood coagulation factor viii. Protein Expr Purif.

[26] Rich RL, Myszka DG (2000). Advances in surface plasmon resonance biosensor analysis. Curr Opin Biotechnol.

[27] Zhang J, Li H, Wang X, Qi H, Miao X, Zhang T (2012). Phage-derived fully human antibody scfv fragment directed against human vascular endothelial growth factor receptor 2 blocked its interaction with vegf. Biotechnol Prog.

[28] Lu D, Shen J, Vil MD, Zhang H, Jimenez X, Bohlen P (2003). Tailoring in vitro selection for a picomolar affinity human antibody directed against vascular endothelial growth factor receptor 2 for enhanced neutralizing activity. J Biol Chem.

[29] Chen R, Li L, Weng Z (2003). Zdock: An initial-stage protein-docking algorithm. Proteins.

